# Vascularized tumor models for the evaluation of drug delivery systems: a paradigm shift

**DOI:** 10.1007/s13346-024-01580-3

**Published:** 2024-04-15

**Authors:** Elliot Lopez-Vince, Claire Wilhelm, Teresa Simon-Yarza

**Affiliations:** 1 Laboratoire Physico Chimie Curie, PCC, CNRS UMR168, Institut Curie, Sorbonne University, PSL University, 75005 Paris, France; 2 Université Paris Cité, Université Sorbonne Paris Nord, LVTS Inserm U1148, 75018 Paris, France

**Keywords:** Tumor-on-chip, In vitro models, Vascularization, Drug delivery systems, Cancer models

## Abstract

As the conversion rate of preclinical studies for cancer treatment is low, user-friendly models that mimic the pathological microenvironment and drug intake with high throughput are scarce. Animal models are key, but an alternative to reduce their use would be valuable. Vascularized tumor-on-chip models combine great versatility with scalable throughput and are easy to use. Several strategies to integrate both tumor and vascular compartments have been developed, but few have been used to assess drug delivery. Permeability, intra/extravasation, and free drug circulation are often evaluated, but imperfectly recapitulate the processes at stake. Indeed, tumor targeting and chemoresistance bypass must be investigated to design promising cancer therapeutics. In vitro models that would help the development of drug delivery systems (DDS) are thus needed. They would allow selecting good candidates before animal studies based on rational criteria such as drug accumulation, diffusion in the tumor, and potency, as well as absence of side damage. In this review, we focus on vascularized tumor models. First, we detail their fabrication, and especially the materials, cell types, and coculture used. Then, the different strategies of vascularization are described along with their classical applications in intra/extravasation or free drug assessment. Finally, current trends in DDS for cancer are discussed with an overview of the current efforts in the domain.

**Graphical Abstract:**

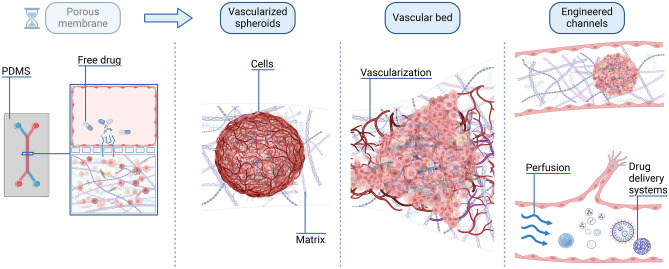

**Supplementary Information:**

The online version contains supplementary material available at 10.1007/s13346-024-01580-3.

## Introduction

The array of strategies to address cancer continues to expand as shown by recent proof-of-concept achievements in CAR-T cell therapy or extracellular vesicles (EVs) treatment within tumoral models [1–3]. Yet, the translation rate from preclinical studies to successful therapies remains low [4]. Besides, these studies are expensive, laborious, and rely on animal models that often present major limitations in faithfully reproducing the pathophysiology of the disease. Notably, cancer animal models have undergone significant advances in the last decades [5]. Among these models, mice have emerged as the predominant choice in cancer pre-clinical research owing to the high homology of their genome to the human genome, easy gene modifications and breeding. Mouse models can be chemically induced, established through injection of cell lines or patient cells to generate xenografts, or genetically engineered. Injection of human cells to better mimic the human disease implies working with immunodeficient mice that fail to reproduce the immune response that has been proved to be a key factor in the evolution of the disease and response to drugs. However, the injection of human cells to better emulate human diseases involves working with immunodeficient mice, which fail to replicate the immune response—a pivotal factor in disease progression and drug response. Genetically modified animal models, while capable of inducing orthotopic tumor formation in immunocompetent mice, often fall short in predicting tumor responses to drugs due to disparities in the immune systems between humans and rodents. In recent years, concerted efforts have been directed towards developing humanized models that replicate the tumor microenvironment and the patient's immune system. Although these models hold promise, their implementation is intricate, requires long times with an increased cost. Additionally, some cases may be prone to graft-versus-host disease [5]. Concurrently, significant progress has been achieved on 3D models that can now integrate several types of cells, a tunable supportive matrix, and fluid compartments [6]. Their preclinical relevance and reliability have been assessed and confirmed [7, 8], laying the groundwork for potential scalability within the industry. Besides, these in vitro models include dynamic interactions between the different compartments, most often by the perfusion of the liquid environment, to constitute a vascularized tumor model (VTM) [9–11]. Indeed, the tumor vasculature plays a pivotal role in essential processes such as immune response, drug delivery [12], or metastasis mechanisms (notably through its influence on the epithelial-mesenchymal plasticity (EMP) [13]).

VTMs constitute an actively investigated domain for which cells, materials, and microfluidic setups are extensively described. Some reviews focused on disease mechanisms and progression events such as metastasis or intra- and extravasation [14, 15]. Limited attention has been dedicated to exploring the effect of drugs on these in vitro models [16], and this number further diminishes when considering drug delivery systems (DDS) [17]. Given that the final aim of in vitro models in tissue engineering is either implantation or use as a biomimetic drug assessment platform, it prompts the question of why so few models are used to investigate the efficacy of drug carriers.

DDS are engineered to carry a drug throughout the body to its intended target, by either passive or active targeting [18]. DDS comprise polymeric and lipid nanoparticles (NPs), EVs, and liposomes as the most classical carriers [19, 20], but also micelles [21], metal–organic frameworks [22], or microbubbles can be used [23]. These carriers can be of primary importance to bring sensitive contents within cells such as proteins or nucleic acids, which are otherwise rapidly degraded in vivo. Besides, they can also increase cell internalization, which is known to be the limiting factor for intracellular delivery and facilitate the targeting of a specific cell population. A growing interest arises for efficient drug carriers that can target specific tissues or cell types, modulate the drug release, or enhance immune stealthing and therefore improve their pharmacokinetic profile. Candidates that fulfill these requirements are likely to lead the innovation in the field, as testified by the FDA approval of anticancer treatment based on delivery platforms, such as Doxil or Abraxane in 1995 and 2005 respectively. The emergence of NPs prepared from pro-drug polymers has made it possible to reduce what have been some of the major limitations of nano-encapsulated drugs for decades, such as poor drug loading, burst release or uncontrolled biodegradation. To that extent, the contribution of Prof. Couvreur in this field deserves mention, and notably his recent works on self-assembled lipid pro-drug NPs based on squalene [24].

Independently of their composition, NP formulation and targeting abilities are often optimized in vitro using classical 2D models before transitioning to animal models. This switch can introduce unpredictable differences in the outcomes, contributing to the failure of DDS evaluated in clinics as compared to the promising candidates observed in fundamental studies. This limitation was already highlighted by Prof. Couvreur, whose efforts in recent years have also been aimed at obtaining more relevant in vitro cancer models for the evaluation of DDS [25]. An example is the pioneer scaffold-free multicellular model of pancreatic cancer made of endothelial cells (ECs), pancreatic cancer cells and fibroblasts (FBs), developed by his team [26].

**Fig. 1:**
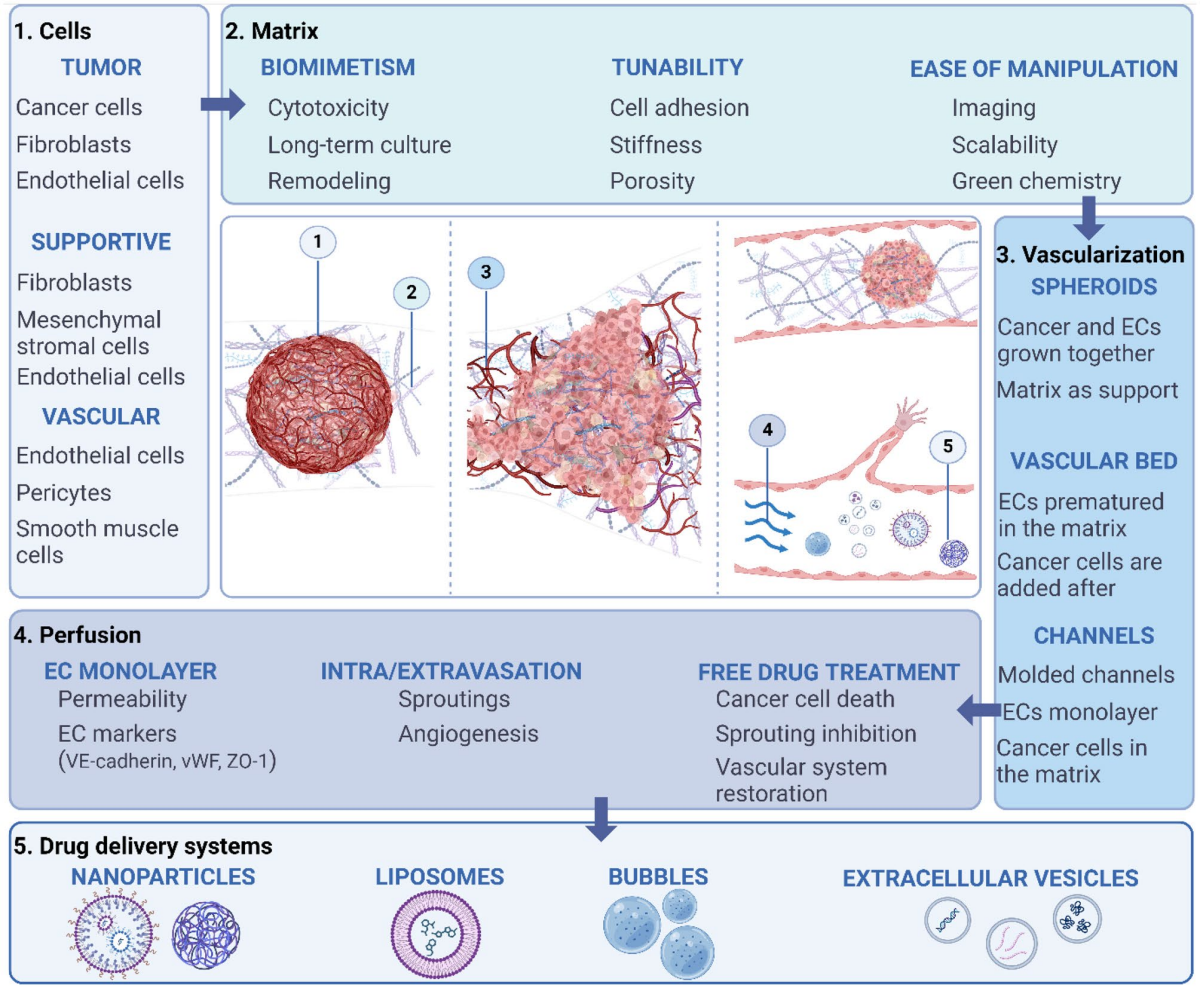
An advanced in vitro vascularized tumor model integrates biomechanical, chemical, and biological features, to mimic the tumor microenvironment. These models can be perfused using differential pressures or microfluidic setups to investigate the efficiency of drug delivery systems for the treatment of cancer. Created with Biorender.com. ECs: endothelial cells; VE-cadherin: vascular endothelial-cadherin; vWF: von Willebrand factor; ZO-1: zona occludens-1

Thus, the goal of this review is to focus on the development and use of VTMs as platforms to assess DDS. To that extent, principal characteristics of VTMs existing in the literature are first detailed including support matrix, cell types employed, and types of vascularized models (Fig. 1). Then, an in -depth study about the use of such VTMs to evaluate vascular permeability, foster cancer invasion, or assess DDS efficiency in vitro is provided.

## Components of vascularized tumor models: recreating the tumor microenvironment

### The matrix

**Fig. 2:**
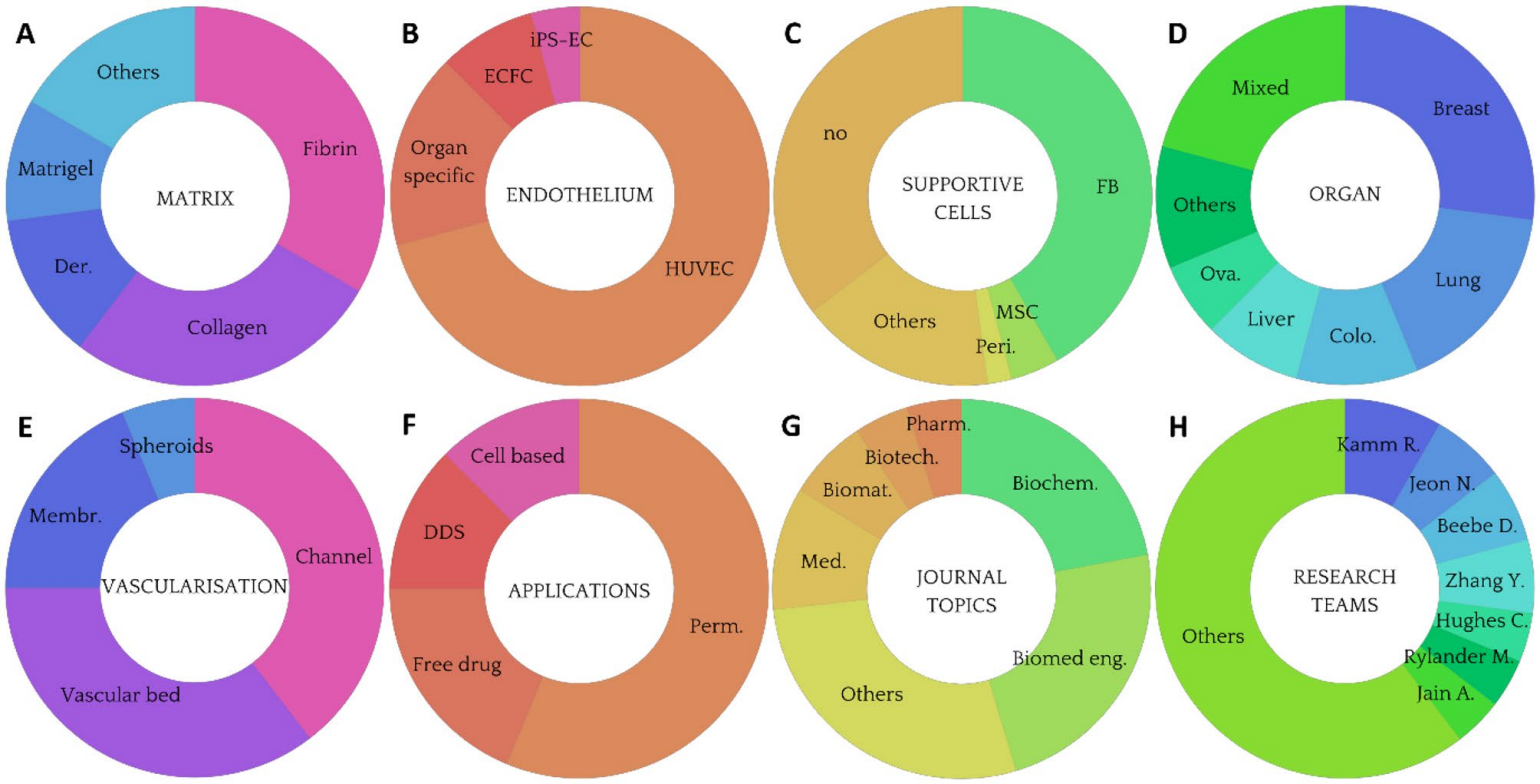
Quantitative analyses of the different setups used for VTMs on 48 articles assessed (Table S1). **A** Materials used for the matrix, fibrin and collagen being the most common, along with derivatives of these two biomaterials (der.). **B** Type of ECs used for the vascularization, HUVECs being the most used, followed by organ specific ECs, ECFCs, and iPs-ECs. **C** Use of supportive cells like FBs, MSCs, or pericytes (peri.). **D** Organotypic models usually focus on breast, lung, colorectal (colo.), liver, or ovarian (ova.) cancer. **E** These models use channels covered with ECs, vascular bed, or vascularized spheroids (spher.), as well as porous membranes (membr.) setups for their vascularized part. **F** The drug delivery perspectives are evaluated either by a simple evaluation of the vascularization permeability (perm.), or by infusion of free drugs, DDS, or cell-based therapies. **G** The 48 articles selected were published in 27 different journals, which topics have been summarized here. Most proficient journals are specialized on biochemistry (biochem.) and biomedical engineering (biomed. eng.), but also about biomaterials (biomat.), medicine (med.), biotechnologies (biotech.), or pharmacology (pharm.). More transversal topics are also present, such as soft matter or mechanical engineering, and have been gathered in the category “others”. **H** Published articles were mostly written by research teams in the USA and Korea, but also from China, and The Netherlands. VTMs: vascularized tumor models, ECs: endothelial cells, HUVEC: human umbilical vein EC, ECFC: endothelial colony forming cells, iPS-EC: ECs derived from induced pluripotent stem cells, FBs: fibroblasts, MSCs: mesenchymal stromal cells. Methodology similar to the quantitative analysis realized by Bouquerel et al. [50]

VTMs have benefited from recent progress in microfluidics, biomaterials, and 3D imaging. As for 3D simpler models of cancer, a vital aspect is the possibility of precisely controlling the stiffness and geometry of the matrix, which can impact subsequent characterizations. The choice of the matrix is thus determined by technical constraints, but also by physiological relevance, availability, and ease of manipulation. For these reasons, collagen I and fibrin matrices are, by a large margin, the most used compounds in the literature (Fig. 2A).

As it is the main component of the extracellular matrix (ECM), collagen I exhibits a physiologically relevant stiffness range, and it allows cell degradation, which is key for angiogenesis and cancer invasion. Furthermore, cells can adhere to this matrix, thus avoiding the need for additional coating steps during material preparation. Collagen I has been widely used in in vitro models, for example to create micro-vessels supported by pericytes to study the angiogenic and thrombotic behavior of the system [27], to investigate the effect of the vessel network on cancer cell migration [28], or to assess the delivery of NPs in one of the first reported VTMs used for DDS in 2014 [29]. As its use became more standardized, its versatility was exploited across a spectrum of concentrations, ranging from 1.5 mg/mL to 7 mg/mL [30–32]. Such variation impacted the stiffness and thus migration properties of encapsulated cells. For example, Ozkan et al*.* reached compression modulus values of 0.9–1.9 and 4–6 kPa by adjusting the concentration from 4 mg/mL to 7 mg/mL, mimicking healthy and tumorigenic liver conditions respectively [32]. The mechanical properties of the collagen matrix have been shown to also depend on the acidic solvent used to rehydrate the collagen [33], which must therefore be detailed in all protocols. Along with hydrochloric acid, acetic acid is one of the most commonly used. The resulting collagen solution can be directly mixed with cells [30, 34], although in most models, cells are seeded after the complete gelation of their matrix [35, 36].

The other gold standard is fibrin, which is bio-sourced and highly tunable due to its two-component composition that offers great control over its gelation process and mechanical properties. Fibrin requires a solution of fibrinogen and thrombin to be mixed to trigger the gelation process. The cell solution can be added to either thrombin [37] or fibrinogen [38, 39], homogenized, and finally the two solutions are combined, rapidly poured into the mold, and incubated for gelation. Notably, Park et al*.* first produced spheroids composed of cancer cells, FBs, and ECs, and after maturation resuspended these spheroids in the thrombin solution [37]. In another model based on fibrin, the transport of anticancer drugs such as paclitaxel was shown to be hindered by the presence of leaky microvascular networks as opposed to a direct treatment of spheroids [40]. It was hypothesized that the density of cells and ECM components in their fibrin matrix containing stromal cells could have curbed the diffusion of drugs, and therefore reduced the spheroid uptake.

Other biomimetic biomaterials, such as Matrigel, have been reported in the literature. Although its biological features support rapid growth of cancer cells such as ovarian, pancreatic, or breast cancer cells [41–44], Matrigel does not allow precise control of the matrix composition. Matrigel was one of the first materials of biological origin to functionalize PDMS chips [42]. In a recent VTM, a PDMS channel coated with gelatin was seeded with ECs while the cancer chamber was filled with Matrigel and patient-derived organoids [43]. These are examples of classical systems that have the advantage of relying on techniques that are used for decades, enabling rapid production of microfluidic chips to assess anticancer drugs efficiency or drug carriers’ performance. However, Kwak et al*.* showed that Matrigel leads to EC channels collapse after 1 day of culture [44]. Therefore, most recent systems try to find alternatives to PDMS, which is too stiff for the cells [45], and Matrigel, whose influence on cells is laboring to analyze due to a complex and often unpredictable composition.

Additionally, basement membrane extract (BME) and decellularized matrix have also been used to build VTMs [46]. A model designed by Liu et al*.* allowed the study of HUVEC angiogenic sprouting in a BME matrix and their promotion by cancer cytokines [47]. The cancer paracrine influence needs to be further investigated with a quantitative analysis, along with a study of the resulting gene regulation among HUVECs. Besides, limited details are provided concerning their in vitro blood vessel, and notably no PDMS functionalization for EC culture is detailed. This raises questions about the phenotype and organization of HUVECs in the PDMS channel. Also, immunostaining of endothelial markers to characterize the monolayer is missing. In line with this, a “blood vessel” bio-printed with a coaxial nozzle to print core and shell layers was fully characterized, including immunostaining marking of CD31 and mRNA expression of endothelial tight junctions [48]. The sprouting of large metastatic cancer spheroids was observed when grown in close contact with the vascularization. The same team also demonstrated that vascular integrity and monocyte recruitment were fostered by the presence of cancer cells, and that this effect was tuned by the distance between cancer spheroids and endothelium [48]. This distance is thus of primary importance when designing a VTM where cancer modules are supposed to be perfused throughout the vascularization.

### The supportive cells

Since the first organ-on-chip models a decade ago [49], tumor-on-chip models have quickly raised and been developed to study the influence of the tumor microenvironment (TME) on its development, drug sensitivity, or metastasis ability [50]. The simplest way to recreate this particular environment is to supplement cells with factors to stimulate vasculogenesis. Indeed, in most studies involving VTM, EC culture medium is enriched with VEGF, bFGF, EGF, and/or ANG1. Recent progress in mechanobiology sheds light on the mechanical stress that the ECM exerts on tumor cells, triggering metastatic behaviors or increasing chemoresistance [51]. The interplays between cancer and supportive cell types that surround them have also been investigated. Notably, stromal cells such as FBs and especially cancer-associated fibroblasts (CAFs) are known to modify the fate of tumors nearby [37, 52]. Recent progress in microfabrication and microfluidics have allowed coculture with spatial control over the different cell populations to explore these interplays. For example, FBs have been shown to have a synergistic effect with flow on the sprouting of ECs [53].The shear stress induced by the interstitial flow was shown to stimulate single cell migration against its direction, while FBs’ cytokines induced the formation of continuous capillaries. Interstitial flow and FBs combined were thus leading to the formation of sprouting against the direction of the flow that showed no leakage when perfused with fluorescent dextran. Besides, this study pinpoints the importance of using organotypiccells instead of generic lineages, as they used primary human lung FB and cancer cells. Other teams henceforth used human lung FB when working on lung-on-chip models [40, 54]. VTMs with tissue specific-ECs such as human breast tumor associated ECs (HBTEACs) have been reported to mimic in vivo interplays between ECs and cancer cells [42, 55]. This was supported by another study that compared the vascular networks formed by both organ-specific and generic ECs in presence of associated cancer cells [8, 34] (Fig. 2B).

Most vessel-on-chip models including VTMs aim at refining the vascularized compartment of the model. To this extent, recent studies suggest that ECs alone have limited angiogenic power, and that the vascularized network quickly retracts without further stimuli [56]. To better mimic the in vivo situation, a coculture with FBs, mesenchymal stromal cells (MSCs), or pericytes is thus preferable. For example, significant differences in angiogenesis were highlighted for a vascular network alone or supported by pericytes [27]. Adding pericytes to improve the vascular development has also been reported in a perfused glioblastoma model previously established by Jung et al*.* that used primary cells, and by Salmon et al*.*, that preferred induced pluripotent stem cells (iPSCs) derived-pericytes [57–59]. Likewise, recent studies in which mature spheroids were integrated to a vascular bed most often used a coculture of FBs, ECs, and cancer cells to foster the connection of the spheroid to the vascular network [37, 54, 56, 60, 61]. Of note, a study established that with thrice the quantity of cancer cells as compared to ECs and FBs, spheroids were seamlessly integrated with the vascular network and exhibited robust growth and viability [60]. In addition to investigating cell ratios, the 3D organization of cells within spheroids has also been examined. A study showed that FBs rapidly reorganized to go in the bulk while cancer cells enriched the shell, which was interpreted as the result of a competition between the different cell adhesion molecules [61]. Such triculture of FBs, ECs and cancer cells have shown the best results in terms of subsequent binding to the vascular network. By using RFP-labeled ECs embedded in the spheroid and GFP-labeled ECs for the vascular bed, Park et al*.* showed how intertwined the red and green networks were after maturation, with heterotypic spheroids showing improved penetration of both nutrients and chemotherapies [37]. Finally, efforts to integrate CAFs instead of generic FB lineages might be beneficial as they are known to tune metastasis and inflammation. For example, a VTM included them along with immune cells to witness the cellular interactions in absence or presence of drugs [52].

In addition to the aforementioned support cells, other types of cells are occasionally integrated in VTMs (Fig. 2C). Notably, Saha et al*.* evaluated the extravasation process of activated platelets under the action of cytokines such as IL6, IL8, CCL2, and TNFα, that were overexpressed by ovarian cancer cells [62]. By doing so, the therapeutic potential of statins was evidenced. Statins contributed to preserve the endothelial adherens junctions, thus impairing the platelets extravasation and reducing subsequent metastasis. The model was later refined by replacing HUVECs with ovarian ECs to extend the study to the influence of platelets in cancer development and metastasis [35]. Human MSCs are also present in the TME and have therefore been integrated in a microfluidic model with breast cancer cells and HUVECs [63]. A significant increase in the development of a robust vascular network was witnessed with this coculture in neutral, bone-, and muscle-mimicking environments. Three years before, the same team had also evaluated the influence of macrophages on the extravasation of cancer cells throughout the secretion of cytokines such as TNFα (Fig. S1B) [64]. This is one of the few studies that included immune cells, although these cells are known to be predominant around an active tumor [65]. Finally, smooth muscle cells are overlooked despite being valuable candidates to encompass the blood vessel complexity. As far as we know, no VTMs include this type of cell so far.

### The tumor compartment

To mimic the tumor, most models are based on immortalized cancer cell lines, broadly available and of well-known genetic origins. Such lineages might not fully recapitulate the in vivo situation, and some models try to include organ specific or even primary cells. For example, primary human MSCs have been used in a breast cancer model to study cancer metastasis [63]. Silvestri et al*.* compared the vascularization of both human and murine primary breast cancer organoids and reported that cancer cells intravasate and perturb the endothelial integrity more frequently in the bulk of organoids as compared to edges [66]. They mention that they obtained these results with HUVECs and that complementary experiments with primary breast ECs would be required. Of note, breast is the most studied system, with a wide range of cells and matrices used [31, 36, 52, 55, 67–69] (Fig. 2D).

Primary cells from colorectal cancer have been used in a quite simplistic model to study the heterogeneities in drug treatment response [70, 71]. Other teams had also developed similar models using colorectal cancer cells and EC lines [34, 38, 47]. On the other hand, using primary cells allowed building a VTM that closely recapitulated the physio-pathological conditions to decipher disease’s progression and outcome, as underlined in a recent lung model [57]. Other teams decided to focus their lung models on its mechanical function to reproduce at best the in vivo situation [46, 53], whereas more simplified models have been used to study the influence of DDS [72] or to increase the throughput for example [40, 73].

The brain is a highly vascularized organ, and its associated diseases are likely influenced by the state of its inner vascularized system. Thus, brain tumors are interesting candidates for VTMs [27, 58, 59]. Other organs with a peculiar organization and function require a balance between simplifying the system and keeping its most relevant characteristics. It is notably the case of the ovaries, where the liquid TME often triggers aggressive behaviors [40, 41, 62, 74]. Concluding, with the development of microfluidics, the serial branching of several organ-on-chip to constitute a “body-on-chip” has been realized to explore the interplays between interconnected organs both in healthy and tumoral conditions [75–77]. For such applications, elastomeric tubing in PDMS that can be covered with ECs to mimic a vascular network have been published few years ago and potentially represent a great improvement [78].

### Characterization

Current trends in VTMs notably consist in complexifying the TME by playing with either the cellular or ECM compartment. Concerning the cellular compartment, an increasing variety of cell types are used, which requires characterizing the stakeholders. For that, the most straightforward technique consists in imaging samples using immunofluorescence in both static and dynamic setups. However, 3D constructs often require building thick samples in which cancer cells, and blood vessels are embedded. Confocal imaging, which is still the most widely used technique for characterization and analysis, only has a penetration depth of about 200 µm due to absorption and scattering [79]. Although imaging techniques have greatly improved in the last decade, and two-photon or light-sheet microscopes are becoming more popular, this technical issue remains a challenge for 3D tissue models development [80]. After cell recovery by scaffold digestion or chip opening, flow cytometry and FACS can be used to study the different cellular phenotypes [52], which can be combined with single cell analyses, such as scRNAseq or velocity monitoring, to get a comprehensive view of the cellular interactions at play [52, 65]. In combination with genomic tools, it can be used to decipher the genetic changes caused by coculture for example [81]. In the EC subpopulation, permeability assays are key to assess the cohesiveness of the endothelial layer and can be associated with qualitative evidence of cellular junctions, such as VE-cadherin and zona occludens-1 (ZO-1). Besides, quantitative assessment of sprouting length and diameter completes the characterization of the newly formed vascular network [53, 81, 82]. For the tumor compartment, adapting protocols from in vivo studies to 3D models by assessing the tumor size and growth under treatment in vitro shows the versatility of VTMs and can ease comparisons between in vitro and in vivo [53]. Finally, analyses of the circulating factors using immunoassays give insights on synergistic or antagonist effects of proteins secreted by the different cell types [34, 37, 83]. 

Besides the cellular compartment, the matrix itself must be characterized to ensure a comprehensive description of the TME. This is key for 3D printing setups, where the mechanical properties of the bioink must be assessed and optimized [48, 84]. Yet, bulk properties of the materials used in the capillary bed process are of importance, with Pradhan et al*.* demonstration of the increase of a PEG-fibrinogen Young’s moduli by parallel-plate compression testing when adding fibroblasts for example [55]. Screening of the matrix composition can also be realized when combining several components as it can impact the performance of cancer cells and ECs [81]. Finally, immunostaining of the different ECM components including collagens I and IV, laminin, and fibronectin can be realized. Of note, second-harmonic generation is a powerful modality that eases the detection of fibrillar collagen and can help distinguish it from exogenous collagen used for the matrix [43].

## Vascularized tumor models: types and applications

### Strategies of vascularization

**Fig. 3:**
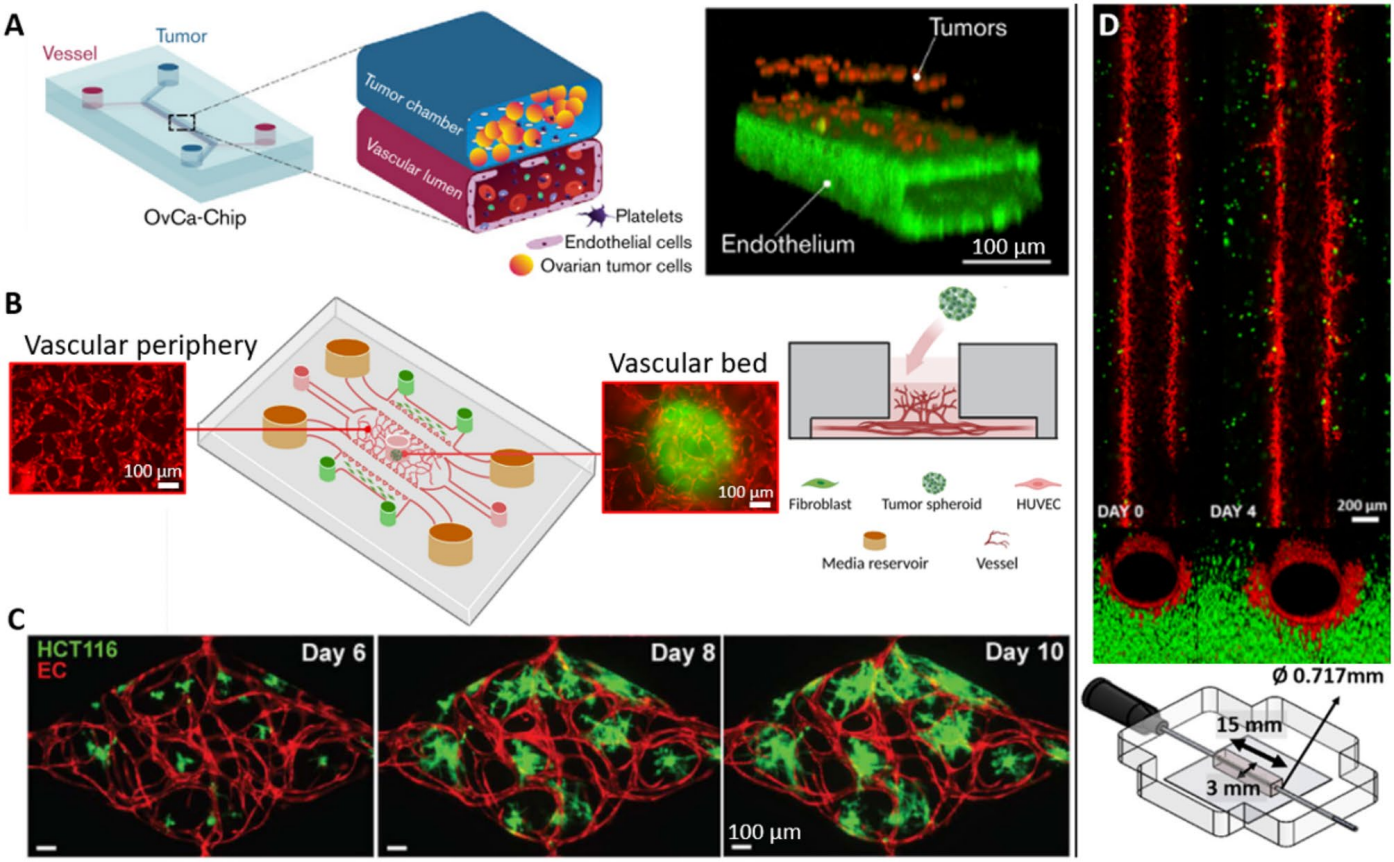
Different strategies of vascularization. **A** Two channels are superimposed and separated by a porous membrane, on which ECs are seeded. Cancer cells are resuspended in a gel phase in the other channel to study the exchanges between the two channels. [62]. **B** A vascular bed composed of ECs homogenized in collagen is formed, and a preformed tumor spheroid is then added on top [54]. **C** Here, cancer cells and ECs are mixed in fibrin to investigate the simultaneous growth of tumors and vascular network [38]. **D** A channel is molded with a needle, which is then removed to enable the vascularization of the channel. After 4 days, ECs have already begun to sprout in the matrix [67]. EC: endothelial cell

VTMs benefit from microfabrication and microfluidics outbreaks to integrate a vascular network to the initial cancer model, to perfuse nutrients or drugs to the system. For that, first VTMs used two superimposed PDMS channels separated by a porous membrane [49] (Table 1). One of the channels was coated with fibronectin and layered with ECs to form a tubular structure after maturation, while the other was most often loaded with a solution of hydrogel and cancer cells, potentially supported by stromal cells as discussed previously. The formation of the endothelial interface was then evaluated by immunofluorescence and permeability assays, and drugs were perfused (Fig. 3A). Such pioneer models relied on stiff PDMS chips, lacked a real 3D organization, and prevented any cell–cell direct interaction because of the presence of a physical barrier between the two cell compartments. Some studies similarly used simple setups to focus on the cellular complexity of the TME [36, 62, 64], or on their application for DDS instead [42, 85]. Apart from that, three different strategies of vascularization can be identified, based on vascular beds, spheroids, or channels covered with ECs (Fig. 2E).

**Table 1:** Main features of studies based on the “membrane” technique

**Technique**	**Matrix**	**Coating**	**Cancer cell** **(10**^**6**^ **cells/mL)**	**Organ**	**EC** **(10**^**6**^ **cells/mL)**	**Supportive cell (10**^**6**^ **cells/mL)**	**Culture conditions**	**Time**	**Flow**	**Ref**
PDMS	2 mg/mL collagen I	poly-D-lysine	3 M/mL HepG2	liver	1 M/mL HUVEC	NA	1:1 DMEM: EGM2 + 20 ng/mL VEGF + 20 ng/mL bFGF	14 days	passive	[28]
PDMS	3–6 mg/mL collagen I	NA	1–100 M/mL MCF7	breast	10 M/mL HMEC	NA	NA	3 days	active	[29]
PDMS	1.5 mg/mL collagen I + 4 U/mL thrombin + 5 mg/mL fibrinogen	1 mg/mL poly-L-lysine + 0.1% v/v glutaraldehyde	20 M/mL MDA-MB-231	breast	20 M/mL HUVEC	NA	EGM2	6 days	NA	[30]
PDMS	2.5 mg/mL collagen I	100 µg/mL collagen I + 30 µg/mL fibronectin	0.5 M/mL SKOV3	brain	0.5 M/mL HOMEC	NA	RPMI 1640	3 days	active	[35]
PDMS	6 mg/mL collagen I + 3 mg/mL Matrigel for channel	1 mg/mL poly-D-lysine	0.05 M/mL MDA-MB-231	breast	2 M/mL HUVEC	0.75 M/mL MSC	EGM2-MV	5 days	NA	[36]
PDMS	Matrigel	50 µg/mL fibronectin	Spheroids (5000 SKOV3 in ULA 96w)	brain	100 M/mL HUVEC	NA	EGM2	2 days	active	[41]
PDMS	Matrigel	100 µg/mL fibronectin + gelatin solution	MDA-MB-231 or MCF7, no concentration	breast	5 M/mL HBTAEC	NA	Endo cell medium	5 days	active	[42]
PDMS	2.3 mg/mL collagen I	10 µg/mL fibronectin	1 M/mL BT474 or MCF7	breast	5 M/mL HUVEC	0.25 M/mL HS578T	MEM + 0.28% NaHCO3 or EGM2	5 days	active	[52]
PDMS	100 µg/mL collagen I + 30 µg/mL fibronectin	100 µg/mL collagen I + 30 µg/mL fibronectin	0.1 M/mL SKOV3	ovarian	5 M/mL HUVEC	200 M/mL primary platelet	EGM2	7 days	active	[62]
PDMS	2.5 mg/mL collagen I	NA	0.8 M/mL MDA-MB-231 or HT1080	breast	2 M/mL HUVEC or primary MVEC	NA	EGM2-MV + 20 ng/mL EGF	3 days	NA	[64]
PDMS	4 mg/mL Matrigel	30 µg/mL fibronectin	1–2 M/mL MDA-MB-231	breast	10 M/mL HVMEC	1 M/mL MSC-derived CAF or NHLF	1:1:1 EGM2-MV:DMEM:EMEM	7 days	NA	[69]
PDMS	2 mg/mL collagen I	1 mg/mL poly-D-lysinehydrobromide + 35 µg/mL collagen I	2 M/mL A549	lung	2 M/mL HUVEC	NA	1:1 EGM2-MV:DMEM	4 days	NA	[72]
PDMS	2.5 mg/mL collagen I + 2.5 mg/mL fibrinogen + 0.5 U/mL thrombin	NA	4 M/mL SKOV3, MKN74, SW620	ovarian, stomach, colorectal	5 M/mL HUVEC or HDLEC	8 M/mL NHLF	EGM2	2 days	NA	[74]
PDMS	Matrigel + 100 ng/mL VEGF	NA	10 M/mL HCT116	colorectal	10 M/mL HCoMEC	NA	Endo cell medium	5 days	active	[85]
PDMS	Fibrinogen + 0.5 U/mL thrombin	NA	Spheroids (5000 1:1 or 4:1 HepG2:HUVEC in ULA 96w)	liver	5 M/mL HUVEC	5 M/mL NHLF	EGM2	6 days	NA	[86]
PDMS	0.2 mg/mL collagen I + 0.5 U/mL thrombin + 2.5 mg/mL fibrinogen + 0.15 U/mL aprotinin	NA	Spheroids (5000 MSCs + 5000 FBs + 2500 HUVEC in ULA 96w)	bone	5 M/mL HUVEC	NA	EGM2	14 days	NA	[99]
PDMS	2.5 mg/mL fibrinogen + 0.5 /mL thrombin + 0.15 U/mL aprotinin	NA	1 M/mL MDA-MB-231 or U87	breast, brain	3–9 M/mL HUVEC	7 M/mL NHLF	EGM2	8 days	NA	[100]

*PDMS* polydimethylsiloxane, *VEGF* vascular endothelial growth factor, *ULA 96w* ultra-low adherence 96-well plates, *EC* endothelial cell, *HUVEC* human umbilical vein EC, *HMEC* human mammary EC, *HOMEC* human oral mucosal EC, *HBTAEC* human breast tumor-associated EC, *HMVEC* human microvascular EC, *HDLEC* human dermal lymphatic EC, *HCoMEC* human colonic microvascular EC, *MSC* mesenchymal stromal cell, *CAF* cancer-associated fibroblast, *NHLF* normal human lung fibroblast, *BFGF* basic fibroblast growth factor, *EGF* epidermal growth factor, *NA* not available

#### Vascular beds

A first approach relies on vasculogenesis to produce a *vascular bed* (Fig. 3B & Table 2). By mixing ECs with the hydrogel solution before gelation, this technique ensures good homogenization and spontaneous formation of micro-vessels with diameters of 10–50 µm on average [54], which would be very challenging to reach by bioprinting or microfabrication. Additionally, the gel solution can also harbor supportive cells to enrich the TME, which constitutes a straightforward protocol to coculture CAFs, MSCs or immune cells for example. In such VTMs, cancer cells can be pre-matured in spheroid elsewhere, or added to the gel solution before crosslinking, which gives a completely random cell repartition where vascularization and tumors develop in parallel [38] (Fig. 3C). On the other hand, growing a disorganized vascular network for too long without flow nor cancer cells lead to vessel retractation, as highlighted by Bonanini et al*.* [56]. This technique can also be combined with pre-vascularized cancer spheroids to help its integration into the vascular bed and ease its perfusion.

**Table 2:** Main features of studies based on the “vascular bed” technique

**Technique**	**Matrix**	**Coating**	**Cancer cell** **(10**^**6**^ **cells/mL)**	**Organ**	**EC** **(10**^**6**^ **cells/mL)**	**Supportive cell (10**^**6**^ **cells/mL)**	**Culture conditions**	**Time**	**Flow**	**Ref**
PDMS	15 U/mL thrombin + 5 mg/mL fibrinogen + 0.125 mg/mL fibronectin	1 mg/mL laminin	0.1 M/mL HCT116	colorectal	10 M/mL HUVEC or ECFC	7 M/mL NHLF	EGM2	10 days	NA	[8]
PDMS	1.5 mg/mL collagen I + 1% carboxymethylcellulose	NA	5 M/mL MCF7	breast	10 M/mL HUVEC	1 M/mL ADSC	EGM2	10 days	NA	[31]
PDMS	4 U/mL thrombin + 6 mg/mL fibrin	5 mg/mL polydopamine	0.15 M/mL in SpheroFilm for spheroid formation	liver, lung	10–15 M/mL HUVEC	NA	EGM + 50 ng/mL VEGF	6 days	NA	[37]
PDMS	3 U/mL thrombin + 10 mg/mL fibrinogen	1 mg/mL laminin	0.2 M/mL SW620, SW480, HCT116, MCF7, MDA-MB-231, MNT-1	colorectal, breast, melanoma	5 M/mL ECFC	5 M/mL NHLF	EGM2	7 days	passive	[38]
PDMS	2 U/mL thrombin + 10 mg/mL fibrinogen + 2.5 mg/mL Matrigel	NA	10 M/mL MCF7, MDA-MB-231, Caco-2	colorectal, breast	10 M/mL ECFC	20 M/mL breast CAF	EGM2	7 days	passive	[39]
PDMS	2 U/mL thrombin + 3 mg/mL fibrinogen	NA	Spheroids (2500–5000 SKOV3 or A549 in ULA 96w)	lung, brain	6 M/mL HUVEC	1.2 M/mL NHLF	VascuLife	7 days	NA	[40]
Polystyrene	0.5 U/mL thrombin + 2.5 mg/mL fibrinogen	NA	1–3 M/mL A549, BxPC3, HepG2, LoVo, SKOV3, MCF7, U87, LNCaP	various	6 M/mL HUVEC	2–6 M/mL lung FB	NA	3 days	NA	[45]
PDMS	BME	NA	5000 ACC or SCC/chamber	colorectal	HUVEC, no concentration	NA	Endo cell medium	2 days	NA	[47]
PDMS	1 U/mL thrombin + 3 mg/mL fibrinogen + 0.15 U/mL aprotinin	NA	Spheroids (10 M/mL Eca-109 mixed 3:1 with NHLF in Matrigel droplets)	esophagus	10 M/mL HUVEC	10 M/mL NHLF	EGM2 or DMEM	15 days	active	[54]
Organo Graft	4 mg/mL collagen I	NA	Spheroids (20′000 hepatocytes (non cancer) + 1000 HUVEC in ULA 96w)	liver	10 M/mL HUVEC	NA	MV2 + 50 ng/mL VEGF + 20 ng/mL bFGF + 2 ng/mL PMA + 500 nM S1P	15 days	passive	[56]
Organo Graft	4.5 mg/mL fibrinogen1 U/mL thrombin	NA	Primary alveolar epithelial cells (non cancer), no concentration	lung	3–4 M/mL HPMEC	1 M/mL primary FB + 0,1 M/mL primary pericytes	1:1 5%LM0002 Lifeline: H6621 Cell Biologics + 85 ng/mL rVEGF + 100 ng/mL rAng-1 + 0.075 U/mL aprotinin	14 days	active	[57]
PDMS	2 U/mL thrombin + 3 mg/mL fibrinogen	NA	Spheroids (5000 4:5 GBM22:pericytes in ULA 96w)	brain	iPS-EC, no concentration	Pericytes, astrocytes	VascuLife + 10% FBS + 2% L-glutamine + 50 ng/mL VEGF-A	7 days	NA	[59]
PDMS	0.5 U/mL thrombin + 2.5 mg/mL fibrinogen + 0.15 U/mL aprotinin + 0.2 mg/mL collagen I	NA	Spheroids (0.02 M/mL MCF7, MDA-MB-231, HepG2, SW620 in ULA 96w)	colorectal, breast, liver	0.5 M/mL HUVEC + in spheroids	NHLF, no concentration	EGM2	7 days	active	[60]
PDMS	2 U/mL thrombin + 3 mg/mL fibrinogen	NA	Spheroids (0.05 M/mL MCF7 or Eca-109 + FB 1:1 in ULA 96w)	breast, esophagus	7 M/mL immortalized HUVEC	1 M/mL commercial lung and primary FB	VascuLife	11 days	NA	[61]
PDMS	2 U/mL thrombin + 2.5 mg/mL fibrinogen	NA	0.4 M/mL MDA-MB-231	breast	20 M/mL HUVEC	0.4 M/mL primary MSC	EGM2-MV + 50 ng/mL VEGF + 100 ng/mL Ang-1	4 days	active	[63]
PDMS	2 U/mL thrombin + 10 mg/mL fibrinogen	NA	5 M/mL primary or SW480, SW620, HPAC, PANC-1	colorectal, pancreas	ECFC, no concentration	NHLF, no concentration	EGM2	16 days	NA	[65]
PDMS	1% w/v µM RGD-Alginate with 200 µM RGD	NA	5 M/mL MCF7, MDA-MB-231	breast	16.7 M/mL OEC	16.7 M/mL human mammary FB	EGM2-MV	14 days	NA	[68]
PDMS	3 U/mL thrombin + 5 mg/mL fibrinogen	1 mg/mL laminin	0.1–0.2 M/mL HCT116 or SW480	colorectal	7 M/mL ECFC	7 M/mL NHLF	EGM-2	10 days	passive	[70]
PDMS	3000 U/mL thrombin + 10 mg/mL fibrinogen	1 mg/mL laminin	2 M/mL primary tumor cells	colorectal	7 M/mL ECFC	7 M/mL NHLF	EGM-2	10 days	passive	[71]
Coverslip	3 mg/mL fibrinogen + 1.2 U/mL thrombin	NA	Spheroids (5000 MDA-MB-231:HUVEC 4:1–19:1 in ULA 96w)	breast	0.25–2 M/mL HUVEC	0.25–2 M/mL human dermal FB	EGM2-MV	7 days	NA	[81]
Well plate	1.6 mg/mL collagen I + 1.12% w/v methylcellulose + 25% v/v Matrigel	NA	220 spheroids of 1000 B16-F10/M10 cells/mL	melanoma	2.2 M/mL MagEC	NA	OptiMEM	3 days	NA	[82]
PDMS	2.5 mg/mL collagen I + 3 mg/mL fibrinogen + 1 U/mL thrombin	NA	Spheroid (5000 H1355 in ULA 96w)	lung	12 M/mL HUVEC	2 M/mL NHLF	VascuLife	7 days	NA	[95]
PDMS	3 U/mL thrombin + 10 mg/mL fibrinogen	1 mg/mL laminin	0.2 M/mL HCT116	colorectal	50 M/mL ECFC	50 M/mL NHLF	EGM2	7 days	passive	[104]
Polystyrene	0.5 U/mL thrombin + 2.5 mg/mL fibrinogen	NA	Spheroid (5000 U87 in ULA 96w)	liver, brain	HUVEC, no concentration	1 M/mL NHLF	EGM2	5 days	NA	[105]
PDMS	2 mg/mL collagen I + 1 U/mL thrombin + 10 mg/mL fibrinogen + 0.15 U/mL aprotinin	25 µg/mL fibronectin	Spheroid (1000 A549 in ULA 96w)	lung	10 M/mL HUVEC or HAMEC	5 M/mL NHLF	EGM2	7 days	active	[107]
PDMS	5% gelMA + 4% gelatin	NA	0.3 M/mL MCF7	brain	0.3 M/mL HUVEC	0.4 M/mL MRC5	NA	7 days	active	[111]
PDMS	5 U/mL thrombin + 10 mg/mL fibrinogen	BME + 1 mg/mL laminin	Conditioned medium from HCT116	colorectal	10 M/mL HUVEC	5 M/mL NHLF	EGM2	7 days	passive	[112]

*PDMS* polydimethylsiloxane, *RGD* arginyl-glycyl-aspartic acid, *gelMA* gelatin methacrylate, *BME* basement membrane extract, *ULA 96w* ultra-low adherence 96-well plates, *EC* endothelial cell, *FB* fibroblast, *NHLF* normal human lung FB, *HUVEC* human umbilical vein EC, *ECFC* endothelial colony-forming cell, *HPMEC* human pulmonary microvascular EC, *iPS-EC* EC derived from induced pluripotent stem cells, *OEC *outgrowth EC, *HAMEC *human microvascular EC, *ADSC* adipose-derived stem cell, *CAF* cancer associated FB, *MSC* mesenchymal stromal cell, *VEGF* vascular endothelial growth factor, *BFGF*: basic fibroblast growth factor, *PMA*: Phorbol 12-myristate 13-acétate, *S1P* D-erythro-Sphingosine 1-phosphate, *Ang-1* angiopoietin-1, *NA* not available

#### Spheroids

*Spheroids* have been extensively used in cancer models because of their good biological relevance, possibility of high throughput production, and relative ease of manipulation for further experiments or analyses [82] (Table 3). Tumor spheroids have been refined to integrate more cell types, such as CAFs and ECs, which allows them to better recapitulate the TME [26, 83]. For example, Ahn et al*.* evidenced that spheroids composed of HepG2 and HUVECs showed more expression of EMP-associated proteins than homotypic spheroids, which correlates with an increased aggressiveness [86]. Usually produced using ultralow attachment plates [60] or hanging droplets [82], they are then transferred to a matrix potentially preloaded with vascular cells. Cancer cells can spread and migrate in this new matrix [54, 87], while the preexisting vascular network can thrive and connect to the microcapillaries inside the spheroid bulk [37]. Alternatively, vascularized spheroids can be studied without any vascularized bed to work on how cells spread on an avascular substrate [81], or how the 3D organization affects their survival [88]. Thus, this strategy focuses on the establishment of a controlled intra-tumoral organization that is then challenged as soon as the spheroids are transferred to the hydrogel compartment. Of note, this strategy is not intrinsically compatible with perfusion and requires another vascularization technique for DDS evaluation.

**Table 3:** Main features of the study that used the “spheroids” technique

**Technique**	**Matrix**	**Coating**	**Cancer cell** **(10**^**6**^ **cells/mL)**	**Organ**	**EC** **(10**^**6**^ **cells/mL)**	**Supportive cell (10**^**6**^ **cells/mL)**	**Culture conditions**	**Time**	**Flow**	**Ref**
PDMS	7% gelMA	NA	1 M/mL SK-BR-3	breast	iPS-EC in spheroids, no concentration	/	McCoy 5A	5 days	active	[83]

*PDMS* polydimethylsiloxane, *gelMA* gelatin methacrylate, *iPS-EC* EC derived from induced pluripotent stem cells, *NA* not available

#### Engineered channels

**Fig. 4:**
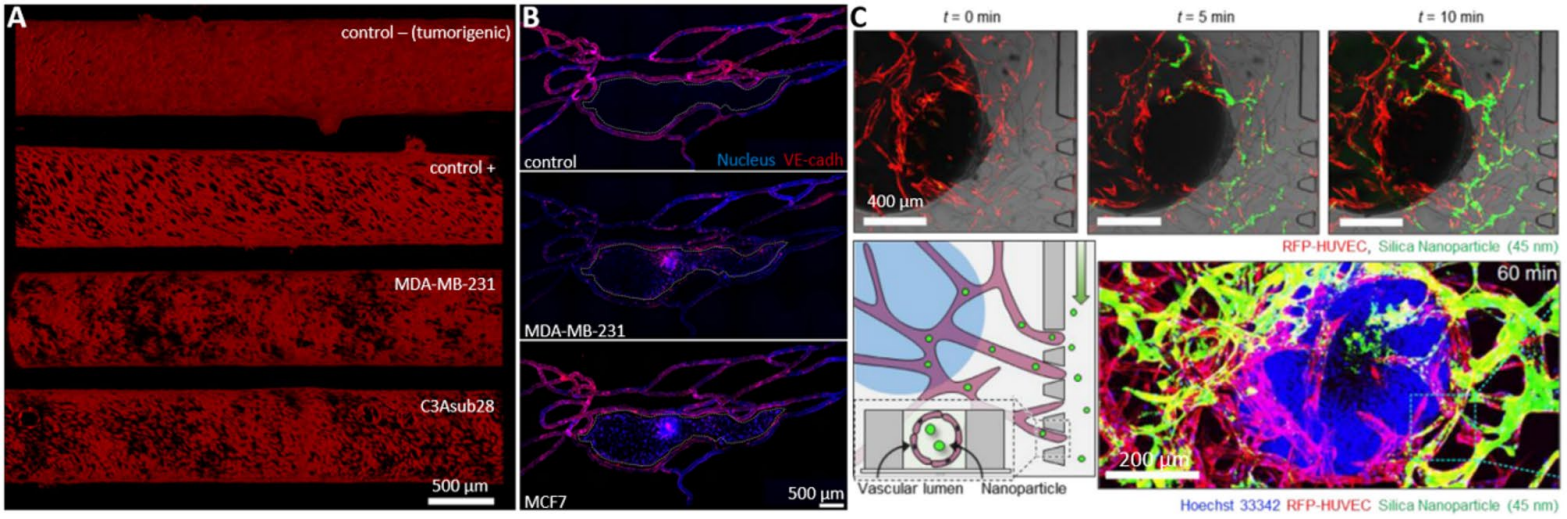
Effects of EC and cancer cell coculture. **A** When cultured with tumor-conditioned medium (control +) or with cancer cells, EC monolayers tend to lose integrity and therefore the permeability of vascularized channels is increased [32]. **B** This can notably be explained by the downregulation of VE-cadherin, especially when grown with aggressive cancer cells like MDA-MB-231. MCF7 have little influence on VE-cadherin expression [42]. **C** After 6 days of culture, the integration of a cancer spheroid within its vascular bed and its perfusion with fluorescent beads could be observed [86]. EC: endothelial cell

For this reason, some studies give particular attention to the patterning of the vascular network, most commonly by 3D printing or soft lithography, and disseminate cancer cells and eventual supportive cells in the matrix [53, 89]. The formation of *manufactured blood vessels* leads to better control of the shape and properties of the vascular network, as well as easier perfusion capability (Fig. 3D & Table 4). Although such studies remain quite rare, bioprinting is becoming now an established technique with promising results that support the high potential of the technology [27, 90]. Numerous strategies to pre-form channels within biomaterials have been investigated [6]. Cheng et al*.* used bioprinting to build a scaffold with a sacrificial ink that is then removed to unveil hollow channels [91]. It allowed them to create a battery of network architectures that can be used to reproduce difficult organ vascular organization for example. Interestingly, they used bacterial cellulose to engineer their matrix, obtaining a unique material behavior with intertwined fibers that metastatic cells are very likely to sense. Their work has been realized with MCF7, and it would be interesting to study if a different outcome is observed with the higher metastatic MDA-MB-231 line, for example. Another team evidenced that the use of very common stereolithography protocols to obtain sinuous geometries was feasible to produce channels of 100 µm [55], a resolution that allows deepening our understanding of ECs evolution when the network is not linear, causing the shear stress to vary much more. Such small constructs have also been obtained in another study [84], where they printed half-channels matched very precisely to obtain channels with diameters ranging from 10 to 500 µm. Other teams used needles of different diameters to produce channels that undergo different shear stress [32, 46, 92]. Thanks to this setup, Ozkan et al*.* were able to compare the evolution of an endothelial monolayer in two different environments: “control”, where the support ECM is composed of 4 mg/mL collagen I, with a diameter of 430 µm, and “tumorigenic”, with a stiffer matrix composed of 7 mg/mL collagen and a bigger diameter, around 730 µm [32]. Therefore, shear stress goes from 4 dyn/cm^2^ to 1 dyn/cm^2^ between healthy and tumorigenic livers, which accounts for the observed loss of integrity of the EC monolayer nearby tumors (Fig. 4A). This effect was further amplified by the addition of TNF-α, or by the loading of cancer cells in the surrounding matrix. The VTM was then combined with a healthy liver model to show the huge decrease in NPs accumulation when first perfused throughout the liver. They supported this finding by underlining the liver targeting-effect of PEGylation on NPs, which might therefore be detrimental for efficient cancer targeting. For comparison, Gadde et al*.* used a similar technique to obtain a diameter of 720 µm for their channel, giving a physiological shear stress of 0.01–0.1 dyn/cm^2^ with their parameters [67]. Finally, some teams also used retaining rods to create their lumen, using PDMS or other non-adhesive materials [34, 93, 94].

**Table 4:** Main features of studies based on the “engineered channel” technique

**Technique**	**Matrix**	**Coating**	**Cancer cell** **(10**^**6**^ **cells/mL)**	**Organ**	**EC** **(10**^**6**^ **cells/mL)**	**Supportive cell (10**^**6**^ **cells/mL)**	**Culture conditions**	**Time**	**Flow**	**Ref**
PDMS	4–7 mg/mL collagen I	1% polyethyleneimine + 0.1% glutaraldehyde	1 M/mL MDA-MB-231	breast	10 M/mL TIME	NA	EGM2	3 days	active	[32]
Polyester PEG	Matrigel	0.2% w/v gelatin	1.7 M/mL patient-derived HPDAC	pancreas	25 M/mL HUVEC	0.85 M/mL human dermal FBs	EGM2	8 days	passive	[43]
PDMS	2:1 to 1:2 Matrigel/collagen I (Matrigel stock at 9.8 mg/mL)	0.1% poly-L-lysine + 1% glutaraldehyde	Spheroids (3000 MDA-MB-231 + 3000–9000 MSC, HUVEC or NHLF in ULA 96w)	breast	1 M/mL HUVEC	3000–9000 MSC, HUVEC or FB in spheroids	EGM2	6 days	active	[44]
PDMS	3 mg/mL collagen I + 1% decellularized ECM	NA	Spheroids (5000 A549 + 2500–5000 HUVEC + 2500 NHLF in ULA 96w)	lung	7 M/mL HUVEC + in spheroids	2500 NHLF in spheroids	2:1:1 EGM2:MEM:RPMI 1640	7 days	passive	[46]
3D printing	1% w/v decellularized skin with 40% w/v Pluronic in 0.1 M CaCl2 solution	NA	10 M/mL SK-MEL-28	melanoma	10 M/mL HUVEC	NA	EGM2	14 days	passive	[48]
PDMS	2.8 mg/mL fibrinogen + 0.5 U/mL thrombin + 0.3 µg/mL aprotinin + 0.3 mg/mL collagen I	2 mg/mL dopamine chloride	Spheroids (A549 in SpheroFilm, no concentration)	lung	0,015 M/mL HUVEC	6000 NHLF in channel	DMEM	5 days	passive	[53]
PDMS	PEG-fibrinogen + 1–2% w/v PEGDA (250 mg/mL)	100 µg/mL fibronectin + gelatin solution	50 M/mL MCF7 or MDA-MB-231	breast	50 M/mL HBTAEC	10 M/mL BJ-5ta	NA	28 days	active	[55]
3D printing	Matrigel	NA	Spheroids (9000 iPS-brain cells in ULA 96w)	brain	6 M/mL iPS-EC	Pericytes, no concentration	Cerebral diff medium + 100 ng/mL VEGF + vit. A	30 days	active	[58]
PDMS	7 mg/mL collagen I	50 µg/mL fibronectin	2–3 primary mammary tumors organoids/µL	breast	50 M/mL HUVEC	NA	MCDB 131	8 days	active	[66]
PDMS	7 mg/mL collagen I	NA	1 M/mL MDA-MB-231, SUM149, MDA-IBC3	breast	0.2 M/mL TIME	NA	EGM2	21 days	active	[67]
PDMS	2 mg/mL collagen I	NA	2 M/mL A549	lung	1 M/mL HUVEC	NA	EGM2	4 days	active	[73]
3D printing	10% w/v gelMA + gelatin	NA	1 M/mL MDA-MB-231	breast	10 M/mL HUVEC	NA	Endo cell medium	3 days	NA	[84]
3D printing	3–7% w/v gelMA + 1–10% PEGDA/PEGOA	NA	1–50 M/mL MCF7	breast	10 M/mL HUVEC or LEC	NA	NA	7 days	NA	[89]
3D printing	0.45–0.9% w/v bacterial cellulose	50 ng/mL fibronectin	2 M/mL MCF7	breast	10 M/mL HUVEC	NA	EGM2	14 days	NA	[91]
PDMS	2.5 mg/mL collagen I	0.1 mg/mL poly-L-lysine + 1% glutaraldehyde	2 M/mL primary cells	pancreas	3 M/mL HUVEC	NA	EGM2	14 days	passive	[92]
PDMS	6 mg/mL collagen I	1% polyethyleneimine + 0.1% glutaraldehyde	NA	kidney	50 M/mL primary cancer-associated EC	NA	MEM complemented	3 days	NA	[93]
PDMS	4 mg/mL collagen I	NA	1–12 M/mL MCF7	breast	HUVEC	NA	X-VIVO10 + 20% FBS + 0.02 mM folic acid + 0.2 mM myo-inotisol	7 days	NA	[94]
3D printing	10 mg/mL fibrinogen + 0.5 U/mL thrombin	NA	1 M/mL A549 or M4A4	lung	10 M/mL HUVEC	0.1 M/mL human dermal FB	1:1 EGM2:DMEM low serum	16 days	NA	[96]
PDMS	3 mg/mL collagen I + 1 mg/mL fibrinogen	30 µg/mL fibronectin	1.8 M/mL MCF7, MDA-MB-231, Hs578T	breast	15 M/mL iPS-EC	NA	iCell endo	4 days	NA	[97]
PDMS	3–6 mg/mL collagen I + 1 mg/mL fibrinogen + 30 µg/mL fibronectin	2% polyethyleneimine + 0.4% glutaraldehyde	0.25 M/mL MDA-MB-231	breast	20 M/mL LEC	NA	1:1 EGM2-MV:DMEM	5 days	NA	[98]
PDMS	3.5–8 mg/mL collagen I	2% polyethyleneimine + 0.1% glutaraldehyde	250 M/mL 786-O	kidney	50 M/mL HUVEC	NA	EGM2	3 days	NA	[101]
PDMS	10% w/v gelMA + 3 mg/mL collagen I	2 mg/mL dopamine chloride or 0.5 mg/mL fibronectin	10 M/mL SKOV3	ovarian	40 M/mL HUVEC	NA	NA	7 days	NA	[102]

*PDMS* polydimethylsiloxane, *PEG* polyethylene glycol, *PEGDA* PEG diacrylate, *PEGOA* PEG octaacrylate, *gelMA* gelatin methacrylate, *EC* endothelial cell, *FB* fibroblast, *HPDAC* human pancreatic ductal adenocarcinoma, *NHLF* normal human lung FB, *HUVEC* human umbilical vein EC, *iPS-EC* EC derived from induced pluripotent stem cells, *ULA 96w* ultra-low adherence 96-well plates, *TIME* hTert-immortalized EC, *HBTAEC* human breast tumor-associated EC, *LEC* lymphatic EC, *MSC* mesenchymal stromal cell, *NA* not available

### Tumor-vasculature interactions

Once cancer cells and ECs are in contact in VTMs, the reciprocal influence they exert on each other can be deciphered. They obviously interact throughout the cohort of chemokines that they release, as extensively displayed in the literature. Notably, tumor-conditioned media led to substantial increase in vessel permeability in a breast model designed by Tang et al*.* [42] (Fig. 4B). By comparing the effects of highly metastatic MDA-MB-231 and poorly metastatic MCF7, they showed that the extravasation of fluorescent dextran, as well as loaded liposomes was more important in the first case. The same effect is observed when cancer cells are directly put in contact with ECs. Besides, another team demonstrated that tumor secretion also improved immune escape, and vascular development [95]. These findings are supported by a third study that highlights the effect of growth factors on both cancer invasion and angiogenesis [96]. Although their proof-of-concept was done using capsules that released growth factors over time, the effects of secreted factors can be extrapolated, as coculture of FBs and cancer cells are known to produce abnormally high quantities of VEGF among others.

To study the effect of tumor secretions during cancer inflammation, Gadde et al*.* used an channel covered with ECs, and cancer cells suspended in the surrounding matrix [67]. First, they highlighted an increased porosity, especially in metastatic conditions, along with the release of inflammatory and angiogenic factors, such as ANG2, VEGF-A, PDGF-bb, IL6, IL8, and MMP2. Besides, they showed a quantitative increase of the number and length of sprouting in the coculture. Their data thus validate the pro-angiogenic and pro-inflammatory effects of cancer cells, as well as an increased leakiness of the vessels [97, 98]. On the other hand, upregulation of angiogenic genes like VEGF and vWF in ECs and of genes associated with cell proliferation and migration like VIM, LAMB3, and IGFBP5 in cancer cells have been demonstrated [86] (Fig. 4C). Yet, proinflammatory factors could also lead to vascular degradation [53]. By growing cancer cells and a developing vascular network at the same time, tumors seemed very likely to impair vessel formation, which ended up in its retraction after a few days, especially without flow.

The presence of tumors around vascularization can also lead to endothelial reorganization and trigger the formation of mosaic vessels. Although their origin is still discussed, a team managed to recreate in vitro a setup where they observed their formation, along with other rare events, such as vessel constriction or pull [66]. Indeed, cancer spheroids as close as 5 µm from the vessel could insert in the endothelial monolayer to form a mosaic vessel, then giving an easy starting point for cancer extravasation and metastasis. They were also shown to impair the vessels’ function by creating dead-ends or ramifications. Such findings are supported by similar conclusions using primary cells [92]. Such systems could also be coupled with DDS to study the transport of drugs in actively remodeling vessels and their possible outcome.

### Process mediated by tumor vasculature

Developing a VTM aims at understanding the interactions that occur when cancer cells, ECs, and eventual stromal cells converge in a dynamic environment. In most studies, the integrity of the model is first assessed, usually by immunofluorescent imaging of endothelial markers like VE-cadherin, vWF, CD31, and ZO-1. The permeability of the endothelium is then evaluated both in normal and cancer coculture conditions by perfusing the system with fluorescent beads, or FITC-dextran of different molecular weights (classically, 3 kDa and 70 kDa). This is either done using live microscopy or regular confocal acquisitions, depending on the setup (Fig. 2F).

Numerous articles focus on metastasis and therefore assess the intra- and extravasation potential of cancer cells in their circulating environment. For that, some teams created a vascularized environment in which circulating cancer cells were added [99] while others suspended them in a gel [100]. With this latter model, Lee et al*.* highlighted the modulatory effect of VEGF on cancer angiogenesis, and of TNF-α on intravasation. Similarly, a bone-mimicking setup with cancer cells embedded in a fibrin matrix was developed to investigate the activation of Snail by osteo-generated factors like CXCL5 [36]. Due to the relative simplicity of these setups, numerous studies using a similar approach have been published for almost a decade. To increase the relevance of the system, Aleman. et al*.* used microfluidics to generate a multi-organ platform and studied the preferential metastasis sites of colorectal cancer cells in circulation [76]. This model encompasses a global overview of the metastatic process, yet it overlooks the TME in contact with the metastasizing cells. Thus, the process itself can be scrutinized, by looking at the intravasation of cancer cells suspended in a matrix throughout an endothelium, for example by bioprinting cancer spheroids near blood vessels [53]. Alternatively, the extravasation can also be studied by loading cancer cells in the lumen of channels previously layered with ECs, as done in a breast cancer model using an ingenious channel production technique with preproduced sacrificial PDMS rods, called LumeNEXT [97, 101]. Finally, Ozkan et al*.* modeled the whole process by using two successive chips representing a breast tumor with a healthy liver and studied the extravasation of cancer cells from the breast cancer chip to its lumen, and then their intravasation in the liver chip under flow [32].

The above-mentioned systems have also been used to perfuse free growth factors, drugs, or nucleic acid-based therapeutics (Fig. 2F). Although this is out of the scope of this review, it is noteworthy pointing up that several recent works also reported perfusion with immune or CAR-T cells [54, 61, 102]. Indeed, using VTMs as therapy screening platforms is of great relevance as the TME and notably its dynamic aspect is known to affect therapeutics penetration and efficiency [70, 103]. Significant discrepancies between tumor spheroids and VTM drug sensitivity were evidenced by perfusing patient-derived xenografts in a colorectal model with the gold standard treatment FOLFOX, along with the promising TGF-β inhibitor galunisertib [71]. Direct applications of anticancer treatment for evaluation of their potency has also been realized and pinpointed vessel resorption after paclitaxel treatment both for cell lines and patient-derived cells [39]. Assays have also been routinely conducted by Phan et al*.* for numerous anticancer drugs to establish the relevance of their high-throughput platform for drug screening [104]. Another team used a very different setup to focus on MMP9 in cancer spheroids [73]. Treatment efficacy could be established by quantifying the decrease of the vascularized volume for antiangiogenic drugs such as bevacizumab [105], or thanks to the evolution of the tumor volume, which is commonly assessed in vivo and was adapted here for this pancreatic VTM [106].

## Drug delivery development based on vascularized tumor models

The development of DDS relies on costly and laborious in vivo pharmacokinetic/pharmacodynamic evaluations of few candidates that have been selected after formulation optimization. The use of VTMs to assess the integrity of drug carriers, their behavior during perfusion, or their targeting ability could greatly reduce the costs and accelerate DDS commercialization. Besides, studies can be conducted at different scales to decipher DDS fate at the level of the tumor microvasculature and in the ECM. To understand better how drugs penetrate within compact tumors and how the TME affects the cell sensitivity, in vitro 3D models are paving the way for translational studies. In our opinion, the evaluation of anticancer drug carriers in vascularized models should be more ambitious and include more innovant DDS strategies currently proposed in the literature. In this section, the studies in which VTMs are used to evaluate DDS, mainly nanoformulations, are discussed (Table 5). In the coming years, VTMs should be applied to other systems for which understanding how they cross the endothelium and navigate within the tumor mass to reach their target is essential to evaluate their potential clinical efficacy, such as plant-based formulations, nanocrystals, extracellular vesicles, carbon nanotubes, dendrimers, micelles etc.

**Table 5:** Characteristics of the DDS assessed in VTMs and associated main outcomes

**VTM**	**Organ**	**Carrier System**	**Targeting**	**Drug**	**Outcome**	**Ref**
vascular bed	breast	NP made of fullerene core + lipid outer shell embedded in mesoporous silica	NA	doxorubicin	16-fold increase in efficacy as compared to free drug	[31]
membrane	ovaries	PEG- or folic acid-modified liposomes and PLGA NP	NA	NA	Combined effect of gel and endothelial cells act as a double barrier that better mimics the in vivo biodistribution and diffusion	[41]
membrane	breast	Liposomes of HSPC, cholesterol, DSPE-PEG2000	E-selectin, ICAM-1	NA	Significant increase of the targeting using coated liposomes as compared to plain ones, but no improvements brought by the dual coating	[42]
channel	lung	Liquid metal particles made of eutectic gallium-indium, DSPC, DSPE-PEG2000	NA	doxorubicin	Qualitative loading of embolism particles in capillaries that were not reached by perfusion but challenging quantitative assessment	[53]
vascular bed	brain	Liposomes of DSPC, DSPE, DSPG, with polyelectrolyte multilayer shell of poly(l-arginine) + propargyl-modified poly-(l-aspartic acid)	Angiopep-2	cisplatin	Similar tumor size decrease as compared to free drug, but more effective and more selective cancer cell killing	[59]
vascular bed	lung	Exosomes produced by HEK293T and ultrafiltrated before loading	NA	miRNA-497	Reduced A549 migration and VEGF-A production, inhibition of VEGFR-2 expression in HUVECs	[72]
spheroids	breast	NP of magnetite in SiO2 shell functionalized with Pluronic F127	NA	doxorubicin	Good targeting and effect of the NPs as compared to the free drug, with a different biomarker expression pattern for cardiac functionality	[83]
channel	colorectal	CMCht-PAMAM dendrimer	NA	gemcitabine	Reduced expression of MMP1, Casp-3 and Ki67 as compared to free drug	[85]
vascular bed	lung	Liposomes of DPPC, DSPE-PEG2000, cholesterol, and Top Fluor PC	ICAM-1	paclitaxel	Effective adhesion to inflamed vessels even under flow and with remarkable specificity	[107]
NA	breast	NP of hyarulonic acid, PEG, cholanic acid	NA	doxorubicin	Validation of a VTM model using free drug and drug-loaded NP	[109]

*DDS* drug delivery systems, *VTM* vascularized tumor model, *NP* nanoparticle, *PEG* polyethylene glycol, *PLGA* polylactic glycolic acid, *HSPC* hydrogenated soy L-α-phosphatidylcholine, *ICAM-1* intercellular adhesion molecule-1, *DSPC* distearoylphosphatidylcholine, *DSPE* 1,2-distearoyl-sn-glycero-3-phosphoethanolamine, *DSPG* 1,2-distearoyl-sn-glycero-3-phosphoglycerol, *VEGF* vascular endothelial growth factor, *HUVEC* human umbilical vein endothelial cell, *miRNA* microRNA, *CMCht* carboxymethylchitosan, *PAMAM* poly(amidoamine), *MMP1* matrix metalloproteinase-1, *Casp-3* caspase-3, *DPPC* dipalmitoylphosphatidylcholine, *DOPE-647N* dioleoylphosphatidylethanolamine bound to Atto647, *PPMT* (ω-pentadecalactone-co–N-methyldiethyleneamine-co-3,3′-thiodipropionate, *ECM* extracellular matrix, *SiO2* silica, *NA* not available

### Liposomes

Liposomes have been used as DDS for thirty years, with the FDA approval of Doxil in 1995 or paclitaxel liposomes in 2003. They are still much used today as they hold great advantages such as the ability of shuttling hydrophilic and hydrophobic drugs at the same time by playing with their lipid bilayer, as well as their great versatility for precise targeting. To validate their 3D vascular model, Paek et al*.* designed liposomes coated with anti-ICAM1 antibodies to target the activated ECs [107]. They showed a good liposomal targeting after perfusion of the vasculature with TNFα to trigger ICAM1 overexpression. To try to better depict the gradients of inflammatory factors as observed in vivo, they used lipopolysaccharides beads to foster a local inflammation of their vascular bed. Their liposomes were concentrated in the activated zone, validating a successful targeting of ICAM-1. Similarly, another study showed substantial increases in both adhesion to the vascular compartment and subsequent extravasation to the cancer area by perfusing their VTM with liposomes decorated with anti-E-selectin antibodies (Fig. 4B). Yet, dual targeting focusing E-selectin and ECAM1 showed no improvements as compared to single targeted liposomes [42]. Straehla et al*.* used an angiopep-2 peptide to increase blood–brain barrier (BBB) permeability and thus the delivery of cisplatin by their liposomal shuttles in a model of vascularized glioblastoma [59]. No significant effect of the targeting was evidenced both in vitro and in vivo, leading them to conclude that their model was a relevant mimic of the in vivo situation and could permit drug testing prior to or instead murine studies. Perspectives for refining their model include addition of flow as well as immune cells, along with the coupling with another organ-on-chip device to assess DDS pharmacokinetics throughout the BBB. Interestingly, it was also suggested that stiffer materials seem to be internalized less efficiently when comparing polystyrene and liposomal NPs, although the opposite was asserted for PEG-PLGA NPs as compared to soft PEG-liposomes [41]. This may be caused by the activation of different internalization pathways depending on NP stiffness [108].

### Lipid nanoparticles

**Fig. 5:**
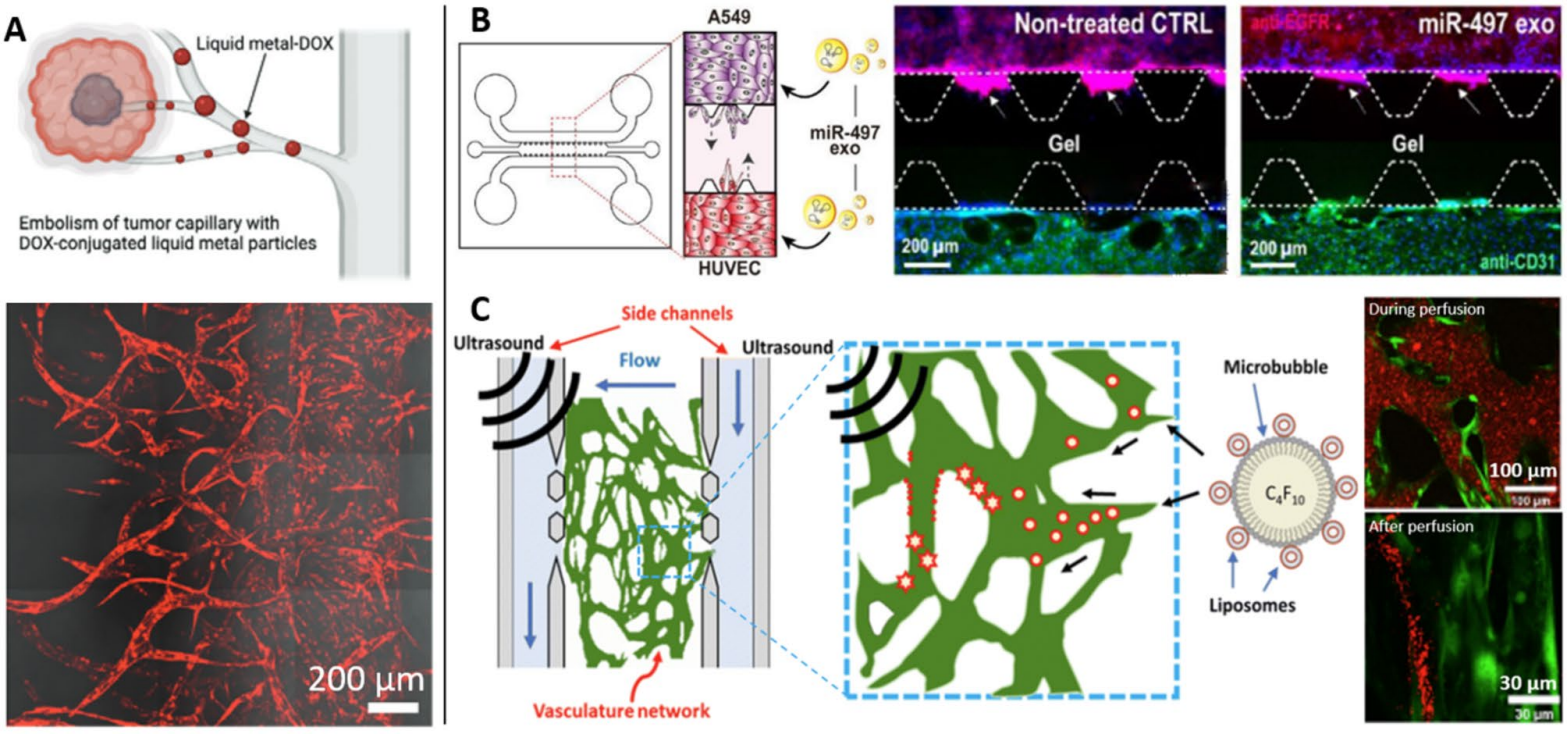
Drug delivery in VTMs. **A** Liquid metal NPs loaded with doxorubicin were injected within the vascularization and successfully circulated through capillaries and around the tumor in 3 days without targeting [53]. **B **A549 and HUVECs were seeded in side channels while collagen I was loaded in the middle. The treatment of exosomes carrying microRNA showed decreased cancer cell invasion (white arrows) as well as inhibition of the endothelial reorganization into tubular constructs [72]. **C** Liposomes were loaded in microbubbles that were collapsed using inertial cavitation induced by ultrasound in the vascular bed, enabling the deposition of the liposomes [112]

Besides liposomes, lipid NPs have hooked huge interest recently and became one of the most used DDS nowadays [31]. Other types of NPs are also widely studied, including polymer-based, graphene oxide-based, or metal compositions. For example, B. Han and colleagues decorated hyaluronic acid NPs with doxorubicin to compare with free drugs and showed that it labored to penetrate in poorly vascularized tumors [109, 110]. The two different pharmacokinetic profiles observed in the study may originate from lengthened circulation time of NPs instead of sustained targeting. This can be explained by a slower diffusion of NPs as compared to free drug, as highlighted in a FB/EC/cancer coculture setup using PEG-PPMT polymeric NPs loaded with docetaxel [111]. Yet, this remark concerns in vitro setups with little to no flow and no targeting. This is likely to be different for more complex models that better depict the in vivo situation, with for example the stealthing brought by DDS to avoid immediate degradation of therapeutics. Yet, circulation of NPs without targeting can be quite long, with liquid metal NPs loaded with doxorubicin showing an effective decrease of the tumor only 3 days after the initial perfusion throughout their vascular network for example [53] (Fig. 5A). Finally, dendrimer NPs loaded with gemcitabine showed a successful release of the drug in a VTM and pinpointed a possible threshold in drug efficacy, giving a stark increase in cell viability after the first hundreds of micrometers of NPs diffusion in the vessel [85].

### Others

Some other strategies stand out, such as the use of EVs or microbubbles to carry drugs or oligonucleotides. Jeong et al*.* used exosomes to deliver microRNA to both HUVECs and cancer cells in a quite simplistic VTM [72] (Fig. 5B). They showed that this microRNA substantially curbed cancer migration, as well as angiogenesis by suppressing VEGF-A and VEGFR2 expression. An ingenious DDS used microbubbles to deliver loaded liposomes at the tumor site, combined with ultrasound both to permeabilize cell membranes and to make microbubbles collapse to release their content [112] (Fig. 5C). They also showed that integrin α_v_β_3_ was upregulated in HUVECs and FBs following tumor conditioned media treatment, and thus they decorated their liposomes with anti-α_v_β_3_ antibodies to target the TME. Increased liposomes accumulation near the tumor was shown, with a significant effect of the ultrasound bursting.

## Deriving insights from interconnected fields

The modeling of vascularized tissues necessitates a multidisciplinary approach (Fig. 2G), and strategies developed for other tissues, both healthy and diseased, can significantly contribute to advancing research on VTMs. The liver plays a pivotal role in drug metabolism, making the development of 3D in vitro hepatic models essential for drug development. This area is of particular interest for the pharmaceutical industry, which invests significantly in research, especially leveraging microfluidic and 3D bioprinting technologies [113, 114]. The latter has been employed to generate liver organoids from patient-derived cells in recent scientific investigations, in collaboration with pharmaceutical industries. The liver VTM employed in these studies comprised a tricellular composition, meticulously organized to mimic an in vivo architecture, thereby providing a representative platform for studying drug-induced liver injury (DILI). Following exposure to various pharmaceutical compounds, this model demonstrated superior fidelity in replicating the human response to drugs at the tissue level when compared to conventional culture methods [115]. Another example is the Emulate Liver-Chip, made of hepatocytes, Kupffer cells, stellate cells, and ECs lining the vascular channel. Utilizing data from this system in pharmaceutical decision-making processes has been proposed as a strategy to mitigate the occurrence of clinical trial failures associated with DILI [116]. An investigational model is also vLAMPS, a biomimetic human liver encompassing the same four cell types, including liver sinusoidal ECs lining the vascular channel, with applications in drug delivery [117]. This device enables the establishment of continuous oxygen zonation, offering significant insights into its role in toxicology and disease progression. Such strategies might be adapted to cancer models to better understand oxygen gradients within tumor masses, influencing drug delivery efficiency. Moreover, the versatility of 3D liver models extends to replicating mechanical properties and biochemical stimuli during fibrosis, as detailed in this comprehensive review [118]. Applying this technology to cancer models, where ECM modifications are pivotal for drug delivery, holds promise for enhancing the efficacy of antitumoral DDS.

Inspiration for optimizing VTMs can be drawn from other tissue models where the vascular component plays a crucial role. Notably, the BBB has undergone numerous advancements, with some currently available as commercial products such as SynVivo and Mimetas [119]. These models typically incorporate various cell types, including astrocytes, vascular ECs, and pericytes, and are often integrated with Transendothelial Electrical Resistance (TEER) monitoring systems to assess barrier integrity and permeability. Extensive research with these models has emphasized the superiority of humanized models over rodent models. The use of ECs derived from patients allows for a more accurate recapitulation of in vivo scenarios, especially in dynamic models that provide better predictions of drug passage compared to static models [120]. A particularly intriguing prospect is the adaptation of these BBB models to incorporate the tumor fraction, facilitating the evaluation of DDS for the treatment of brain tumors. Ideally, the development of models capable of replicating distinct disruptions in the BBB based on the tumor type would be highly advantageous [121].

To conclude, progress in vascularized models of other tissues, particularly in BBB and liver, should inspire more physiological VTM. Besides, the emergence of liver models for industrial drug development shows the path to convert basic research in VTM into clinical benefits. In the next section, some of the challenges to this transfer are addressed.

## Perspectives for translational research

The application of vascularized in vitro models in drug development presents numerous advantages over conventional 2D models, particularly within the field of oncology, where it introduces a pivotal therapeutic factor: the passage through the vascular network and penetration into tumors—a prerequisite for treatment success. Despite the escalating number of vascularized 3D models in research, their transition into pharmaceutical industry applications faces several challenges. The substitution of well-established 2D models, with extensively demonstrated limitations, for 3D models necessitates not only relevance for the intended application but also ease of implementation, reproducibility, reliability, and cost-efficiency.

In this context, the complexity of a model in terms of 3D organization, utilization of various cell types, incorporation of primary cells or iPSCs derived from patients, and the application of flow, poses challenges in terms of implementation, standardization, and significantly increased costs [122]. Replicating complex models with a vascular component remains a formidable challenge. The reliability of these models is also compromised, demanding thorough validation before their industrial use. However, a consensus within the scientific community regarding who should assess these models and how they should be evaluated is lacking, as concluded by the European Commission's Joint Research Center based on a 2021 survey by the EU Reference Laboratory for alternatives to animal testing [123].

Furthermore, for drug discovery applications, a crucial scale-up step is absent in most researched models to enable high-throughput screening, using, for example, 384-well plates and systems compatible with standard assays and rapid readouts. Challenges persist in real-time 3D imaging, efficient supernatant collection, and the ability to retrieve cells for further analysis, especially when the therapy under investigation is drug delivery-based: the complexity is even greater in such cases.

Certain biotech companies are actively working to bridge this gap and expedite the integration of complex 3D models into the pharmaceutical industry (Fig. 2H). For example, MIMETAS OrganoPlate® has been specifically designed to incorporate tubules that can be cellularized to mimic blood vessels [124]. Their setup enables co-culture with various cell types without resorting to artificial membranes for assessing cell–cell interactions. More recently, OrganoPlate® Graft has been proposed to position tissue within a microvascular bed, achieving in vitro vascularization for drug administration through the vessel wall [125]. Aimbiotech's organiX Plate also facilitates 3D co-culture, including perfusable vasculature to mimic the TME [126]. While these systems are commercially available and utilized in research, sometimes in collaboration with pharmaceutical companies, they are yet to replace traditional 2D in vitro drug-testing assays.

In summary, progress toward the commercialization of vascularized 3D models remains markedly limited. To pave the way for imminent industrial applications, research should not only focus on enhancing the physiological relevance of these models but also consider their applicability in the industry. Striking a balance between system complexity and industrial utility is essential for achieving meaningful progress in this arena.

### Supplementary Information

Below is the link to the electronic supplementary material.

Supplementary file1 (XLSX 25 KB)

## Data Availability

All data generated or analyzed during this study are included in this published article and its supplementary information files.
